# Non-alcoholic fatty liver disease incidence, remission and risk factors among a general Chinese population with a 6-year follow-up

**DOI:** 10.1038/s41598-018-25641-z

**Published:** 2018-05-15

**Authors:** Jing Wu, Shumei He, Hongqin Xu, Xiumei Chi, Jie Sun, Xiaomei Wang, Xiuzhu Gao, Ruihong Wu, Mingbai Shao, Heng Zhao, Jing Jia, Chunyan Wang, Junqi Niu

**Affiliations:** 1 Department of Gerontology, The First Hospital of Jilin University, Xinmin Street, Changchun, 130021 China; 2 Department of Hepatology, The First Hospital of Jilin University, Xinmin Street, Changchun, 130021 China; 3 Department of Abdominal ultrasound, The First Hospital of Jilin University, Xinmin Street, Changchun, 130021 China

## Abstract

This study aimed to investigate the incidence, remission and risk factors of non-alcoholic fatty liver disease (NAFLD) among a general population with a 6-year follow-up. In total, 691 individuals from the general population in Jilin, China aged 20–75 years participated in two independent cross-sectional surveys carried out in 2007 and 2013. After excluding patients with alcoholism, viral hepatitis and other liver diseases, 646 individuals were finally enrolled in our study. Of the 646 subjects, 512 did not have NAFLD at baseline, while 134 did. Of the 512 individuals without NAFLD at baseline, 188 (36.7%) developed NAFLD during the six-year follow-up period. The baseline body mass index (BMI, OR = 1.49, 1.36–1.64), high-density lipoprotein cholesterol level(HDL-C) (OR = 0.35, 0.16–0.76) and weight gain (OR = 1.22, 1.16–1.29) were independent predictors for NAFLD incidence. Of the 134 subjects with NAFLD at baseline, 33 (24.6%) had no evidence of NAFLD after 6 years. Males (OR = 4.85, 1.98–11.92) and baseline BMI levels (OR = 0.81, 0.70–0.94) were associated with NAFLD remission. Among the general population, the incidence of NAFLD mainly depended on baseline weight and weight gain. Subjects with mild baseline weights and male subjects were prone to NAFLD remission.

## Introduction

Non-alcoholic fatty liver disease (NAFLD) is one of the most common liver diseases, currently affecting 25% of adults worldwide^1^. Although simple NAFLD is usually benign and does not frequently progress to more advanced stages of liver disease^2^, it is an increasing public health concern and has become a major risk factor for cardiovascular disease, cirrhosis and hepatocellular carcinoma (HCC) because of its high prevalence^3,4^. In terms of illness burden, strategies for the primary prevention and treatment of NAFLD are necessary, as they have the potential to save millions of patients’ lives worldwide. NAFLD is a slowly progressive disease, and detailed knowledge about its incidence rates and natural course of progression, which are not fully elucidated, is essential. A follow-up cohort programme is needed to ascertain NAFLD outcomes among the general population, including incidence, remission and related risk factors.

Hamaguchi *et al*. reported that the incidence and remission rates of NAFLD were 10% and 16%, respectively, and found metabolic syndrome to be a strong predictor of NAFLD among a convenience sample of 4401 Japanese employees during only a 1.1 year follow-up period^5^. Sung *et al*. revealed that the development of new fatty liver was associated with increased risks of hypertension in 11,448 patients without hypertension at baseline over a five year follow-up period^6^. During a relative long follow-up period (8.5 years), the Dionysos study reported that ethanol intake was the only risk factor for NAFLD incidence and for the resolution of existing fatty livers^7^. Although the above studies on NAFLD incidence and remission are available, drawing firm conclusions regarding people in the general population remains difficult. We selected subjects who participated in two surveys conducted among the general population in 2007 and 2013 in Dehui, Jilin Province of China. This study aimed to determine the incidence or remission of NAFLD and the related predictive factors during a 6-year prospective follow-up period.

## Results

### Characteristics of the study population

**Figure 1:**
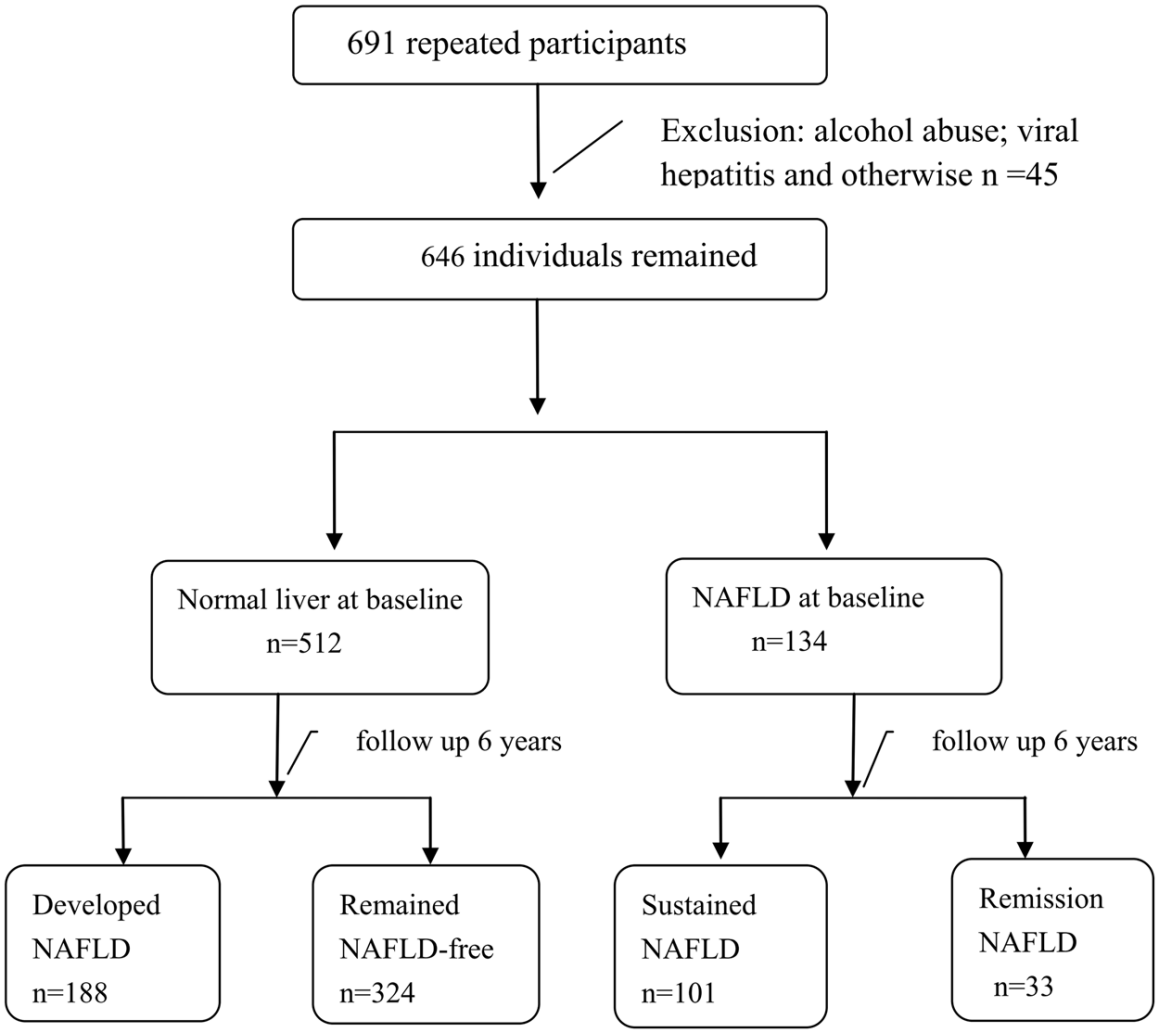
Flow chat of the study population. NAFLD, non-alcoholic fatty liver disease.

As outlined in Fig. 1, 691 subjects participated in both the 2007 and 2013 surveys, and 646 co-participants were enrolled our study after excluding individuals with viral hepatitis (hepatitis B surface antigen (HBsAg) +/anti-hepatitis C virus (anti-HCV) +, n = 34), alcohol abuse (mean ethanol consumption ≧140 g/week in men and ≧70 g/week in women during the past month, n = 10) and other known liver diseases (n = 1). To test for selection bias, the 646 subjects were compared to all subjects from 2007; subjects from the latter population with alcoholism, viral hepatitis and other known liver diseases were also excluded to ensure that the two groups were consistently composed. No differences in the main demographics, anthropometric parameters or biochemical parameters (selected variables are presented in Table 1) were observed between the two groups.

**Table 1:** Comparison of the main baseline characteristics between the repeat population (n = 646) and the total population (n = 3399). Both populations excluded alcoholism, viral hepatitis and other liver diseases in 2007.

Parameter	Repeat population (n = 646)	Total population (n = 3399)	P value
Age (yr)	46.0 (38.0,54.0)	45.5 (37.0,55.0)	0.823
Gender (% male)	42.1%	46.10%	0.064
BMI (kg/m^2^)	23.7 (21.6,26.3)	23.9 (21.5,26.4)	0.708
ALT (U/L)	17.3 (12.8,25.7)	18.0 (12.8,26.3)	0.240
Prevalence of NAFLD on US (%)	20.7%	22.7%	0.303

The results are expressed as medians (25^th^ quartile, 75^th^ quartile) or frequencies (percentage).

BMI, body mass index; ALT, alanine aminotransferase; NAFLD, non-alcoholic fatty liver disease.

Of the 646 repeat participants, 512 (79.3%) did not have NAFLD, and 134 (20.7%) were classified as having NAFLD at baseline (Fig. 1). The characteristics of the repeat population are shown in Table 2. In all patients, the body mass index (BMI), waist circumference (WC), systolic blood pressure (SBP), alanine transaminase (ALT), aspartate transaminase (AST), γ-glutamyl transpeptidase (GGT), total cholesterol (TC), triglycerides (TG) and fasting blood glucose (FBG) parameters increased significantly in all patients, but the low-density lipoprotein cholesterol (LDL-C) and high-density lipoprotein cholesterol (HDL-C) statistics decreased significantly in the 2013 subjects compared to those in the 2007 subjects. The prevalence of diabetes, dyslipidaemia, NAFLD, hyper-TC, hyper-TG and hypo-HDL-C increased significantly, while the prevalence rates of obesity, overweight and hypertension showed slight increases (significance was not observed). However, the prevalence of hyper-LDL-C decreased significantly. In subjects with NAFLD at baseline (n = 134), the BMI and overweight/obesity rates showed slight decreases, though the changes were not significant. The WC, diastolic blood pressure (DBP), and TG measurements as well as the prevalence of hypertension, dyslipidaemia, hyper-TC and hyper-TG were not significantly different among the two groups, and the prevalence of diabetes increased after 6 years. In subjects without NAFLD at baseline (n = 512), the BMI, WC, SBP, GGT, ALT, AST, TC, TG, FBG measurements and the prevalence of overweight, obesity, dyslipidaemia, hyper-TC, hyper-TG and hypo-HDL-C were increased significantly. The prevalence rates of hypertension, diabetes and hyper-LDL-C were not significantly different.

**Table 2:** Characteristics of the repeatedly surveyed populations in 2007 and 2013.

Parameter	All subjects (n = 646)			Subjects with NAFLD at baseline (n = 134)			Subjects without NAFLD at baseline (n = 512)		
	07	13	P value	07	13	P value	07	13	P value
Age (yr)	46 (38,54)	52 (44,60)	/	48 (40,55)	54 (46,61)	/	45 (38,53.75)	51 (44,59.75)	/
Gender (% Male)	271 (42%)	271 (42%)	/	61 (45.5%)	61 (45.5%)	/	210 (41.0%)	210 (41.0%)	/
BMI (kg/m^2^)	23.7 (21.6,26.3)	24.3 (21.9,26.6)	<0.001*	27.3 (25.5,29.3)	27.0 (24.8,28.9)	0.055	22.8 (21.4,25.0)	23.5 (21.6,25.7)	<0.001*
WC (cm)	80 (74,88)	83 (76.5,90)	<0.001*	90 (84,97)	91.1 (87,97.13)	0.058	78 (73,84)	81 (75,87.78)	<0.001*
SBP (mmHg)	125 (115,140)	128 (116,142)	<0.001*	130 (120,150)	138 (126,150)	0.039*	120 (110,135)	124 (110,140)	0.004*
DBP (mmHg)	80 (75,90)	80 (74,90)	0.81	86.5 (80,100)	88 (80,90.5)	0.298	80 (70,90)	80 (72,90)	0.423
GGT (U/L)	18.6 (13.4,33.2)	20 (14,35)	0.035*	31.8 (19.6,53.0)	30 (21,45)	0.024*	16.8 (12.4,28.6)	18 (14,31)	<0.001*
ALT (U/L)	17.3 (12.8,25.7)	20 (14,27)	0.003*	25.35 (16.5,39.0)	22 (17.75,30)	0.002*	16.0 (12.2,23.6)	20 (14,26)	<0.001*
AST (U/L)	21 (17.6,25.7)	24 (21,29)	<0.001*	24.2 (18.8,30.1)	26 (22,31)	0.01*	20.7 (17.6,24.5)	24 (21,28)	<0.001*
TC (mmol/l)	4.24 (3.71,4.91)	4 (4,5)	<0.001*	4.79 (4.16,5.49)	5 (4,6)	0.005*	4.08 (3.65,4.72)	5 (4,5)	<0.001*
LDL-C (mmol/l)	2.9 (2.6,3.4)	2.64 (2.12,3.21)	<0.001*	3.4 (2.8,3.83)	2.85 (2.27,3.35)	<0.001*	2.9 (2.5,3.3)	2.59 (2.1,3.20)	<0.001*
HDL-C (mmol/l)	1.3 (1.2,1.6)	1.3 (1.0,1.5)	<0.001*	1.3 (1.1,1.5)	1.1 (0.9,1.4)	<0.001*	1.36 (1.2,1.6)	1.3 (1.1,1.5)	<0.001*
TG (mmol/l)	1.2 (0.84,1.81)	1.59 (1.09,2.20)	<0.001*	1.84 (1.31,2.72)	2.02 (1.42,2.99)	0.166	1.06 (0.78,1.53)	1.46 (1.05,2.03)	<0.001*
FBG (mmol/l)	4.73 (4.38,5.20)	5.20 (4.80,5.70)	<0.001*	5.08 (4.62,5.52)	5.5 (5.0,6.5)	<0.001*	4.68 (4.34,5.10)	5.1 (4.7,5.5)	<0.001*
Diabetes, n (%)	32 (5.0%)	59 (9.1%)	<0.001*	16 (11.9%)	30 (22.4%)	0.034*	16 (3.1%)	29 (5.7%)	0.066
Hypertension, n (%)	227 (35.1%)	251 (38.9%)	0.185	70 (52.2%)	78 (58.2%)	0.39	157 (30.7%)	173 (33.8%)	0.316
Obesity, n (%)	79 (12.2%)	93 (14.4%)	0.401	56 (41.8%)	47 (35.1%)	0.204	23 (4.5%)	46 (9.0%)	0.01*
Overweight	229 (35.4%)	235 (36.4%)		62 (46.3%)	61 (45.5%)		167 (32.6%)	174 (34.0%)	
Dyslipidaemia, n (%)	320 (49.5%)	393 (60.8%)	<0.001*	109 (81.3%)	106 (79.1%)	0.759	211 (41.2%)	287 (56.1%)	<0.001*
Hyper-TC, n (%)	111 (17.2%)	145 (22.4%)	0.021*	45 (33.6%)	41 (30.6%)	0.695	66 (12.9%)	104 (20.3%)	0.002*
Hyper-LDL-C, n (%)	177 (27.4%)	128 (19.8%)	0.002*	71 (53.0%)	32 (23.9%)	<0.001*	106 (20.7%)	96 (18.8%)	0.48
Hyper-TG, n (%)	183 (28.3%)	284 (44.0%)	<0.001*	75 (56.0%)	86 (64.2%)	0.212	108 (21.1%)	198 (38.7%)	<0.001*
Hypo-HDL-C, n (%)	71 (11.0%)	162 (25.1%)	<0.001*	21 (15.7%)	58 (43.3%)	<0.001*	50 (9.8%)	104 (20.3%)	<0.001*
NAFLD, n (%)	134 (20.7%)	289 (44.7%)	<0.001*	134 (100%)	101 (75.4%)	/	512 (0%)	188 (36.7%)	/

The results are expressed as medians (25^th^ quartile, 75^th^ quartile) or frequencies (percentage).

NAFLD, non-alcoholic fatty liver disease; BMI, body mass index; WC, waist circumference; SBP, systolic blood pressure; DBP diastolic blood pressure; TC, total cholesterol; LDL-C, low-density lipoprotein cholesterol; TG, triglycerides; HDL-C, high-density lipoprotein cholesterol; FBG, fasting blood glucose; ALT, alanine aminotransferase; AST, aspartate aminotransferase.

*Indicates statistical significance.

### Factors predicting NAFLD incidence

**Figure 2:**
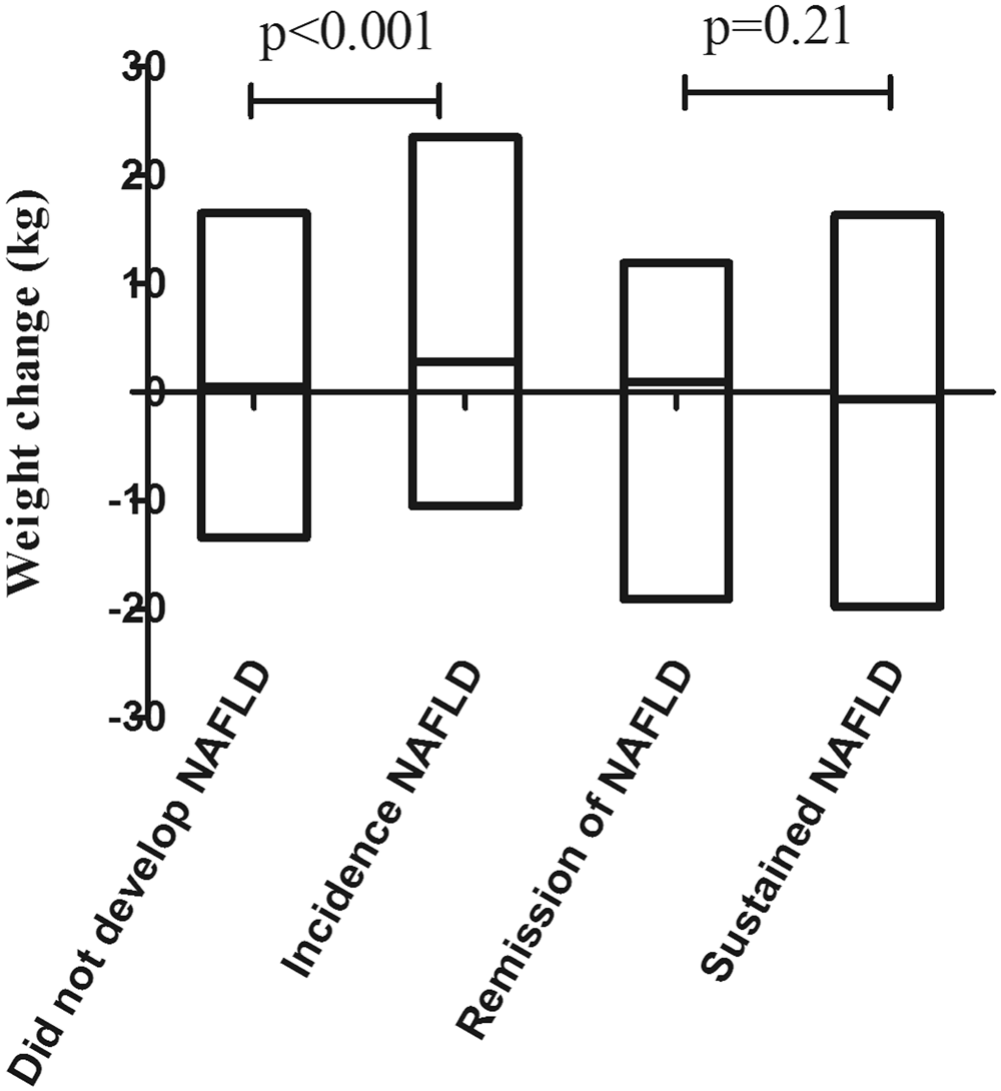
Weight changes in subjects with NAFLD incidence vs. subjects who did not develop NAFLD (based on the cohort with normal livers at baseline, n = 512) and remitted NAFLD patients vs. subjects with sustained NAFLD (based on the cohort with NAFLD at baseline, n = 134). The results are expressed as median values (25^th^ quartile, 75^th^ quartile); NAFLD, non-alcoholic fatty liver disease.

In a sub-analysis of the 512 subjects without NAFLD at baseline, the NAFLD incidence rate at the 6-year follow-up was 36.7% (188/512, annual incidence: 6.1%). The baseline characteristics of the incidence group and the group remaining free from NAFLD are shown in Table 3. Subjects with NAFLD at the 6-year follow-up had higher baseline BMI, WC, TG and LDL-C measurements. The obesity prevalence of the subjects (188) who developed NAFLD was more than eight times that of the other group (324) at baseline (10.1% vs. 1.2%, p < 0.001, Table 3). Notably, the subjects with NAFLD gained significantly more weight than those who did not have NAFLD (2.8 (0, 5.1) vs. 0.5 (1.5, 2.7), Fig. 2). In multivariate logistical regression analysis, the baseline BMI, HDL-C and weight gain measurements remained significantly and independently associated with NAFLD incidence after adjusting for age and gender (Table 4).

**Table 3:** Comparison of baseline parameters between subjects with NAFLD and subjects who did not develop NAFLD (n = 512).

Parameter	Incidence (n = 188)	No incidence (n = 324)	P value
Age (yr)	45.5 (37.0,52.0)	45 (37.8,54)	0.778
Gender (% male)	80 (42.6%)	130 (40.1%)	0.641
BMI (kg/m^2^)	24.7 (22.8,26.4)	22.1 (20.4,23.8)	<0.001*
WC (cm)	83.0 (78.0,88.0)	75.0 (71.0,81.0)	<0.001*
SBP (mmHg)	120 (110,135)	120 (110,130)	0.265
DBP (mmHg)	80 (75,90)	80 (70,85)	0.172
GGT (U/L)	19.1 (13.1,30.7)	16.0 (12.1,25.3)	0.024*
ALT (U/L)	17.3 (12.8,25.0)	15.4 (11.5,22.5)	0.004*
AST (U/L)	20.7 (17.6,25.1)	20.7 (17.6,24.5)	0.841
TC (mmol/l)	4.03 (3.55,4.65)	4.14 (3.68,4.77)	0.366
LDL-C (mmol/l)	2.85 (2.50,3.20)	2.90 (2.57,3.30)	0.411
HDL-C (mmol/l)	1.30 (1.10,1.50)	1.4 (1.20.1.60)	<0.001*
TG (mmol/l)	1.22 (0.88,1.80)	0.98 (0.72,1.46)	<0.001*
FBG (mmol/l)	4.71 (4.37,5.09)	4.66 (4.32,5.12)	0.366
Diabetes, n (%)	9 (4.8%)	7 (2.2%)	0.254
Hypertension, n (%)	63 (33.5%)	94 (29.0%)	0.32
Obesity, n (%)	19 (10.1%)	4 (1.2%)	<0.001*
Overweight, n (%)	93 (49.5%)	74 (22.8%)	
Dyslipidaemia, n (%)	84 (44.7%)	127 (39.2%)	0.228
Hyper-TC, n (%)	27 (14.4%)	39 (12.0%)	0.494
Hyper-LDL-C, n (%)	33 (17.6%)	73 (22.5%)	0.213
Hyper-TG, n (%)	49 (26.1%)	59, (18.2%)	0.043*
Hypo-HDL-C, n (%)	26 (13.8%)	24 (7.4%)	0.021*

The results are expressed as medians (25^th^ quartile, 75^th^ quartile) or frequencies (percentage).

NAFLD, non-alcoholic fatty liver disease; BMI, body mass index; WC, waist circumference; SBP, systolic blood pressure; DBP diastolic blood pressure; TC, total cholesterol; LDL-C, low-density lipoprotein cholesterol; TG, triglycerides; HDL-C, high-density lipoprotein cholesterol; FBG, fasting blood glucose; ALT, alanine aminotransferase; AST, aspartate aminotransferase.

*Indicates statistical significance.

**Table 4:** Factors associated with NAFLD incidence or remission according to multiple logistical regression analysis.

Associated factor	NAFLD incidence				NAFLD remission			
	β	SE	OR (95% CI)	P value	β	SE	OR (95% CI)	P value
Baseline BMI	0.40	0.05	1.49 (1.36–1.64)	<0.001*	−0.21	0.08	0.81 (0.70–0.94)	0.006*
HDL-C	−1.06	0.40	0.35 (0.16–0.76)	0.008*	—	—	—	—
Weight gain	0.20	0.029	1.22 (1.16–1.29)	<0.001*	—	—	—	—
Sex (male)	—	—	—	—	1.58	0.46	4.85 (1.98–11.92)	0.001*

NAFLD, non-alcoholic fatty liver disease; BMI, body mass index; HDL-C, high-density lipoprotein cholesterol.

*Indicates statistical significance.

### Factors predicting complete NAFLD remission

In a sub-analysis of 134 subjects diagnosed with NAFLD at baseline, the NAFLD remission rate during this period was 24.6% (33/134, annual remission: 4.1%). The baseline characteristics of the subjects who achieved or failed to achieve remission are presented in Table 5. The prevalence of obesity in subjects who still had NAFLD was higher than that of subjects in remission, while there were no differences in the prevalence rates of diabetes, hypertension and dyslipidaemia among the two groups (Table 4). In addition, there were no significant differences in weight changes between the subjects still having NAFLD and those in remission (−0.7 (−4.15, 2.2) vs 0.9 (−4.5, 3.25), Fig. 2). Though the subjects in remission maintained the same weight (from 70 (61.5, 77.5) to 70.8 (60.4, 77.9), their TG levels slightly decreased (from 2.14 (1.35, 2.90) to 1.91 (1.31, 2.83), p = 0.743) after 6 years. The baseline BMI measurements and male sex were independently associated with NAFLD remission in multiple logistical regression analysis using a forward (likelihood ratio) approach (Table 4).

**Table 5:** Comparison of baseline parameters between subjects achieving or not achieving remission NAFLD (n = 134).

	Sustained (n = 101)	Remission (n = 33)	P value
Age (yr)	47.0 (39.0,55.0)	49 (44.5,54.0)	0.446
Gender (% male)	37 (36.6%)	24 (72.4%)	<0.001*
BMI (kg/m^2^)	27.7 (25.9,29.7)	25.9 (24.8,28.5)	0.004*
WC (cm)	91.0 (85.0,97.0)	87.0 (82.0,94.5)	0.158
SBP (mmHg)	135 (120,150)	130 (120,140)	0.352
DBP (mmHg)	90 (80,100)	80 (80,90)	0.068
GGT (U/L)	29.9 (19.1,50.5)	35.3 (24.3,62.3)	0.383
ALT (U/L)	27.6 (16.7,41.7)	23.1 (15.4,35.6)	0.278
AST (U/L)	23.8 (19.0,30.1)	24.5 (18.2,29.8)	0.696
TC (mmol/l)	4.80 (4.19,5.50)	4.78 (4.10,5.46)	0.979
LDL-C (mmol/l)	3.40 (2.87,3.90)	3.40 (2.75,3.70)	0.716
HDL-C (mmol/l)	1.30 (1.10,1.50)	1.40 (1.10.1.50)	0.6
TG (mmol/l)	1.78 (1.29,2.63)	2.14 (1.35,2.90)	0.542
FBG (mmol/l)	5.08 (4.62,5.50)	5.04 (4.51,5.57)	0.524
Diabetes, n (%)	14 (13.9%)	2 (6.1%)	0.476
Hypertension, n (%)	55 (54.5%)	15 (45.5%)	0.423
Obesity, n (%)	46 (45.5%)	10 (30.3%)	0.031*
Overweight, n (%)	47 (46.5%)	15 (45.5%)	
Dyslipidaemia, n (%)	83 (82.2%)	26 (78.8%)	0.797
Hyper-TC, n (%)	35 (34.7%)	10 (30.3%)	0.678
Hyper-LDL-C, n (%)	54 (53.5%)	17 (51.5%)	1
Hyper-TG, n (%)	54 (53.5%)	21 (63.6%)	0.322
Hypo-HDL-C, n (%)	16 (15.8%)	5 (15.2%)	1

The results are expressed as medians (25^th^ quartile, 75^th^ quartile) or frequencies (percentage).

NAFLD, non-alcoholic fatty liver disease; BMI, body mass index; WC, waist circumference; SBP, systolic blood pressure; DBP diastolic blood pressure; TC, total cholesterol; LDL-C, low-density lipoprotein cholesterol; TG, triglycerides; HDL-C, high-density lipoprotein cholesterol; FBG, fasting blood glucose; ALT, alanine aminotransferase; AST, aspartate aminotransferase.

*Indicates statistical significance.

Moreover, the weight gain of the subjects with NAFLD at baseline (−0.45 (−4.35, 2.70)) was significantly lower than that of subjects free from NAFLD at baseline (1.35 (−1.00, 3.98)).

## Discussion

In this study, we took advantage of the availability of a relatively large cohort of people from the general population who participated in both surveys. Unlike previous large follow-up studies performed on patients, none of the subjects enrolled in our study abused alcohol, and their liver enzymes were mostly normal^7^.

Our results clearly showed that the prevalence of NAFLD increased from 20.7% to 44.7% after 6 years, which was significantly higher than the world average level^1^. During the 6-year period, 36.7% of the subjects free from NAFLD at baseline ultimately developed NAFLD, as evidenced by regular ultrasonography. This incidence rate was obviously higher than those among the general populations utilized in Zelber-Sagi’s study (20% with 7-year follow up, annual incidence: 2.86%)^8^ and Hamaguchi’s study (8.6%/year)^5^. At first glance, the incidence rate the among general population was expected to be lower than that in the Dionysos study, which did not exclude alcohol drinkers and was performed on patients with elevated liver enzymes (10% incidence rate with 8.5-year follow up)^7^. However, we observed the opposite result, which could have been due to differences in the demographical characteristics, race and gender distributions, or most importantly, in the economic areas. The incidence rate in our study was still higher than that found in a Hong Kong study (13.5% developed NAFLD in 3–5 years) even though the racial characteristics of the two studies were close^9^. However, the incidence rate in our current study was close to that found in a Sri Lankan study (43.4% incidence rate with a 7-year follow up, annual incidence: 6.2%)^10^, which may be related to the fact that both Sri Lanka and China are developing countries.

Because previous studies have demonstrated that both NAFLD and NAFLD incidence are intimately connected with obesity, diabetes and metabolic syndrome^9,11^, it was not surprising that various parameters, such as BMI, LDL-C, and weight gain, were found to be independent predictors for NAFLD in this 6-year follow-up study. Thus, our findings implied that baseline weight and weight gain are more risky for NAFLD incidence than other metabolic risk factors, such as diabetes and hypertriglyceridemia, which may be explained be the fact that overweight/obese subjects have a higher prevalence rate than subjects with a normal BMI^12^. While the importance of obesity for NAFLD development has been demonstrated in previous studies^8,10,13,14^, NAFLD incidence cannot be prevented by regulating body weight within the normal range. Indeed, insulin resistance develops during weight gain even if the subject is within the normal range of weight^15^. Therefore, having a BMI within the normal limit does not guarantee the prevention of NAFLD development^9^, which is improperly called “lean NAFLD”. Younsossi *et al*. found that “lean NAFLD” patients were independently associated with diabetes when compared to lean controls and with female sex, dyslipidaemia and insulin resistance when compared with obese/overweight NAFLD patients^16^.

The high prevalence and incidence of NAFLD highlights its growing burden in developing economies, such as China. Thus, in addition to controlling weight gain, targeting more community-based effective measures to reduce other metabolic risk factors, such as diabetes and dyslipidaemia, appears to be a critical method for decreasing the prevalence and incidence of NALFD. If not controlled, NAFLD is expected to become the most common indication of chronic liver disease^11^.

Regarding NAFLD remission, 24.6% of our study population with NAFLD at baseline were in remission after 6 years (annual remission: 4.1%), which was somewhat lower than the one third of individuals achieving NAFLD remission after 7 years^8^ and the 50% of the study population achieving this status after 8 years^7^ in other studies.

NAFLD is more likely to develop and less likely to regress^5^. As we mentioned that weight gain was a predictor of NAFLD incidence in the previous paragraph, we also investigated whether weight loss was a predictor of NAFLD remission. This view has been confirmed by many studies. Some studies suggested that a 5% weight reduction may be sufficient for improving histological steatosis^17^ and serum ALT levels^18^. Zelber-Sagi *et al*. revealed that a weight loss of 2.7 ± 5.0 kg was significantly associated with NAFLD remission, and a 75% remission rate was observed among NAFLD subjects who lost more than 5% of their baseline weight. Therefore, weight reduction is considered the best available treatment option for NAFLD^8^. However, the regression of NAFLD is not entirely dependent on weight loss. For instance, one study found that serum uric acid levels were associated with NAFLD remission in Chinese males^19^. In addition, Korean data implied that moderate-to-vigorous exercise is beneficial for improving the resolution of an existing fatty liver regardless of BMI changes during the follow-up period^20^. Thus, controlling or reducing the metabolic factors associated with NAFLD development and strengthening exercise regimes should promote NAFLD regression. Our current study showed that some NAFLD subjects in remission did not lose weight during the 6-year period. Indeed, we found that the baseline BMI measurement was associated with NAFLD remission, as the NAFLD subjects with relatively mild baseline weights that maintained their weight throughout the 6-year period had a high probability of NAFLD remission. Our study could not deny that slightly decreased TG levels may have contributed to NAFLD remission to some extent. Additionally, our study results showed that males were prone to NAFLD remission, and the female sex was previously demonstrated to be an NAFLD risk factor in other studies^16,20^. The reason underlying this association may be related to the hormone levels in women. However, the cohort sample size of 134 subjects was relatively small and needs to be expanded in additional studies to validate this result.

Additionally, the subjects with NAFLD at baseline controlled their weight better than others, perhaps because they had better health awareness than subjects without NAFLD at baseline.

To our knowledge, this is the first study on the incidence and remission of NAFLD performed on a representative sample from the general Chinese population. However, this study does have several limitations. First, certain risks for selection bias remained even though the main parameters of the repeat population did not differ from those of the total population.

Second, the presence or absence of NAFLD was diagnosed by ultrasonography, which may be less sensitive than the liver biopsy method if the levels of liver fat are approximately 30%. Moreover, ultrasonography cannot detect inflammation and/or fibrosis. Third, information on exercise and alcohol consumption was obtained only by directly questioning the participants. Fourth, the sample size of 134 NAFLD patients was relatively small, and the exercise time classification was not sufficiently precise.

In conclusion, this cohort study showed that the incidence of NAFLD among a general Chinse population was high during a 6-year follow-up period. NAFLD incidence was associated with baseline BMI, HLD-C and weight gain measurements, and 24.6% of the patients who had NAFLD at baseline had no evidence of the disease after 6 years. Subjects that had mild baseline weights and subjects that were male had the highest probabilities of achieving NAFLD remission.

## Materials and Methods

### Study population and data collection

In both 2007 and 2013, two independent population-based cross-sectional surveys were conducted with a two-stage stratified sampling method to recruit participants aged 20–75 years in Dehui City, which is representative of Jilin province in terms of economic and cultural development, as described in our previous study^21^. The designs of both studies were similar, and 691 individuals who completed the first survey (2007) also participated in the 2013 survey. A total of 646 residents were enrolled in our study after excluding subjects with viral hepatitis and other known liver diseases as well as those on medication for liver disease and those abusing alcohol.

On both occasions, all subjects were fasted overnight (between 12 and 14 h) prior to undergoing a comprehensive medical examination, including a face-to-face interview by a trained interviewer, clinical and anthropometric measurements (weight, height, WC and BP), biochemical and serological tests (liver enzymes, serum lipid profile, FBG, and HBsAg/anti-HCV tests), and liver ultrasound for the diagnosis of NAFLD. Some of the questionnaire items were as follows: (i) Assess your physical activity level (1. never; 2. <2 hour/day; 3. ≥2 hour/day). (ii) Do you smoke? If yes, have you stopped smoking or how long have you smoked? (iii) Do you drink alcohol? If yes, how long have you been drinking alcohol, and how much do you drink per day? (iv) Describe your diet habits, i.e., do you eat salty, greasy or lighter food? The participants were instructed to take off heavy clothing and shoes before the anthropometric measurements were taken. The BMI was equated to body weight in kilograms divided by height squared in metres. BP was measured two times at 2-min intervals after a 10-minute rest, and the SBP and DBP were calculated based on the average of the two readings. Blood examinations were completed by The First Hospital of Jilin University laboratory department. Ultrasonography was performed on all subjects participating in the two surveys by experienced radiologists who were blinded to individual subject data using the same equipment (180 ultrasound machine with a 3.5 MHz probe, GE Health Care, Wilmington, MA, USA). This study protocol was approved by The First Hospital of Jilin University Ethics Committee, and all methods were carried out in accordance with approved guidelines. Moreover, all the participants provided written informed consent.

### Disease definitions

Diabetes was defined as an FPG ≥7.0 mmol/L, a self-reported history, or the use of insulin or oral hypoglycaemic agents^22^.

Hypertension was defined as an SBP ≥140 mmHg, a DBP ≥90 mmHg, a previous diagnosis disease by a physician, and/or the use of an antihypertensive medication within the past 2 weeks regardless of BP readings^23^.

We referred to the Chinese standards for adult obesity as follows: 18.5 kg/m^2^ ≤ BMI <24 kg/m^2^ was considered normal, 24 kg/m^2^ ≤ BMI <28 kg/m^2^ was considered overweight, and a BMI ≥28 kg/m^2^ was considered obese^24^.

Dyslipidaemia was defined as meeting at least one of the following criteria: TC ≥5.18 mmol/L, TG ≥1.7 mmol/L, HDL-C <1.04 mmol/L or LDL-C ≥3.37 mmol/L^25^.

According to criteria from the “Chinese guidelines on non-alcoholic fatty liver disease (2010)”, NAFLD was diagnosed if the following conditions were met: 1) mean ethanol consumption <140 g/week in men and <70 g/week in women during the past month, 2) negative for HBsAg and anti-HCV, 3) fatty liver diagnosed by ultrasound, and 4) no other liver diseases^26^.

### Statistical analysis

Data were collected and analysed using SPSS 20 (SPSS, Inc., Chicago, IL, USA). Continuous variables were tested using the Kolmogorov-Smirnov test for normality, and non-normal data were presented as medians and quartiles. The Mann-Whitney U test and Wilcoxon signed ranks test were used to assess differences between two independent samples and paired samples, respectively. Categorical variables were presented as frequencies (percentages) and analysed using the Chi-squared test. Multivariate logistical regression analysis was used to identify candidate predictors of NAFLD incidence and remission using a forward (likelihood ratio) approach, whereas the goodness-of-fit of the model was assessed by Hosmer-Lemeshow statistics. P values < 0.05 were considered statistically significant.
